# ERK1/2 communicates GPCR and EGFR signaling pathways to promote CTGF-mediated hypertrophic cardiomyopathy upon Ang-II stimulation

**DOI:** 10.1186/s12860-019-0202-7

**Published:** 2019-06-14

**Authors:** Xin Liu, Lin Lin, Qing Li, Yajuan Ni, Chaoying Zhang, Shuguang Qin, Jin Wei

**Affiliations:** Department of Cardiology, The Second Affiliated Hospital of Xi’an Jiaotong University, 157, Fifth West Road, Xi’an, 710004 Shaanxi China

**Keywords:** ERK1/2, CTGF, EGFR, Hypertrophic cardiomyopathy

## Abstract

**Background:**

Hypertrophic cardiomyopathy occurs along with pathological phenomena such as cardiac hypertrophy, myocardial fibrosis and cardiomyocyte activity. However, few of the specific molecular mechanisms underlying this pathological condition have been mentioned.

**Methods:**

All target proteins and markers expression in the study was verified by PCR and western bloting. H9c2 cell morphology and behavior were analyzed using immunofluorescent and proliferation assays, respectively. And, the CTGF protein secreted in cell culture medium was detected by ELISA.

**Results:**

We found that high expression of CTGF and low expression of EGFR were regulated by ERK1/2 signaling pathway during the cardiac hypertrophy induced by Ang-II stimulation. CTGF interacted with EGFR, and the interaction is reduced with the stimulation of Ang-II. ERK1/2 serves as the center of signal control during the cardiac hypertrophy.

**Conclusion:**

The ERK1/2 cooperates with GPCR and EGFR signaling, and promotes the occurrence and development of cardiac hypertrophy by regulating the expression and binding states of CTGF and EGFR. The study revealed a regulation model based on ERK1/2, suggesting that ERK1/2 signaling pathway may be an important control link for mitigation of hypertrophic cardiomyopathy treatment.

## Background

Cardiomyopathy is a group of lesions that cause progressive dysfunction of the heart due to structural changes in the inferior part of the heart and impaired myocardial wall function. Its clinical manifestations include cardiac enlargement, arrhythmia, embolism, and heart failure [1]. Cardiomyopathy refers to myocardial disease with cardiac dysfunction, clinical types of dilated cardiomyopathy (DCM), hypertrophic cardiomyopathy, restrictive cardiomyopathy, arrhythmogenic right ventricular cardiomyopathy, undifferentiated cardiomyopathy, and specific cardiomyopathy, of which DCM is the most common. DCM may be related to myocardial damage caused by abnormal metabolism of viruses and bacterial drugs [2]. Viral myocarditis is considered to be the most important cause. The etiology and pathogenesis of this disease are still unclear. In addition to idiopathic and family hereditary, it is thought to be related to persistent viral infection and autoimmune-induced myocardial damage. The main pathological changes were enlargement of the heart chamber, thinning of the ventricular wall, and often accompanied by mural thrombus [3].

As a major kinase signal molecule in the cell, ERK1/2 plays an important role in numerous intracellular signaling pathways including G protein coupled receptor (GPCR) and receptor tyrosine kinase (RTK), and participates in numerous physiological and pathological events [4]. There also been many reports on the relationship between the related signal pathways in inflammation-related cardiomyopathy and the processes of cardiomyocyte fibrosis [5, 6]. As we all know, ERK1/2 can not only be used as a downstream molecule of the classical epidermal growth factor receptor (EGFR) signaling pathway, but also can be activated by GPCR signaling pathway [7, 8]. However, no research has been conducted on the cooperative regulation between these two signals in cardiomyopathy.

Database analysis showed that EGFR showed low expression in DCM patients, while connective tissue growth factor (CTGF) showed high expression [9]. As a secreted protein, CTGF plays an important role in cardiomyopathy. Previous studies have shown that it has a certain role in promoting myocardial fibrosis and myocardial cell apoptosis [10]. However, studies based on tumor models have shown that CTGF can act as a ligand of EGFR, stimulate EGFR activation, and play a role in promoting tumor cell proliferation and even tumor cell EMT [11]. This differs from the differential expression of CTGF and EGFR in DCM patients. However, their specific regulatory pattern in DCM and relationship with activation of ERK1/2 signaling molecules are unknown.

In this study, we studied the specific regulatory mechanisms in the cardiac hypertrophy cell model, revealed its specific molecular mechanism, and provided a certain theoretical basis and possible molecular targets for the treatment of DCM and other hypertrophic cardiomyopathy.

## Results

### Ang-II stimulation promotes cardiomyocyte fibrosis and hypertrophy

**Fig. 1:**
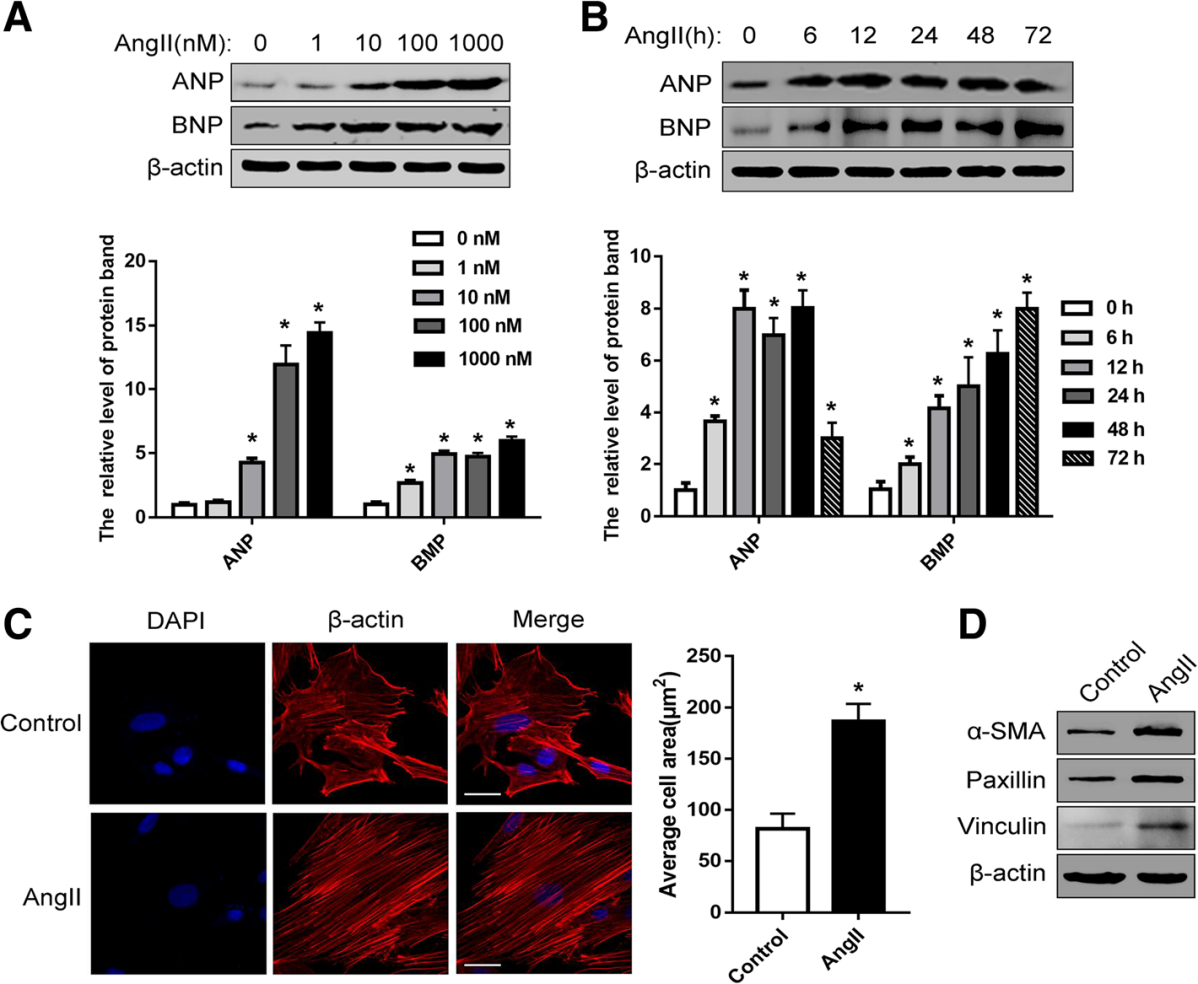
Ang-II Stimulation Promotes Cardiomyocyte Fibrosis and Hypertrophy. **a** Western blot detection for ANP and BNP expression in H9c2 cardiomyocytes with different concentrations or (**b**) time of Ang-II treatment. (**c**) 24 h after seeding H9c2 cardiomyocytes in 10-cm culture plates, 10^− 8^ M AngII was added first. And, the β-actin of H9c2 cardiomyocytes was immunofluorescent stain. **d** Western blot detection for α-SMA, Paxillin and Vinculin expression in H9c2 cardiomyocytes with or without Ang-II treatment. All experiments were repeated at least three times. Data represent mean ± SD; * *P* < 0.05

Ang-II was shown to result in expression of hypertrophic protein markers, including atrial natriuretic peptide (ANP) and B-type natriuretic peptide (BNP) [12]. To evaluate the Ang-II-induced DCM cell model, we verified that Ang-II induces the expression of hypertrophic markers ANP and BNP in H9c2 cardiomyocytes. Experiments showed that the expression of ANP in H9c2 cardiomyocytes began to increase significantly when stimulated with 10 nM Ang-II for 24 h, while BNP exhibited a significant increase tendency at 1 nM Ang-II stimulation for 24 h, and the expression was highest at 10 nM (Fig. 1a). In addition, using 10 nM Ang-II to stimulate H9c2 cardiomyocytes, ANP showed a higher level of expression from 6 to 48 h, and BNP expression was highest at 24 h (Fig. 1b). To further verify the cellular hypertrophy model, we performed an actin staining experiment on H9c2 cardiomyocytes before and after Ang-II induction. The results showed that Ang-II stimulation significantly increased the surface area of H9c2 cardiomyocytes (Fig. 1c).

In addition, myocardial fibrosis is also an important pathological phenomenon of DCM [13]. In the study, protein expression levels of three important cardiomyocyte fibrin markers, α-SMA, Paxillin and Vinculin, were examined. The results showed that all three markers showed significant high expression when induced by Ang-II (Fig. 1d). Although the results of this experiment did not fully explain the occurrence of myocardial fibrosis, it did prove that a similar phenomenon occurred after the treatment. On the other hand, it demonstrated the reliability of the Ang-II-stimulated cell model for induced cardiomyocyte hypertrophy. Taken together above results, 10 nM Ang-II stimulation for 24 h was used as a cell model for cardiac hypertrophy in the subsequent relevant experimental treatment.

### Ang-II stimulation promotes CTGF expression and reduces EGFR expression

**Fig. 2:**
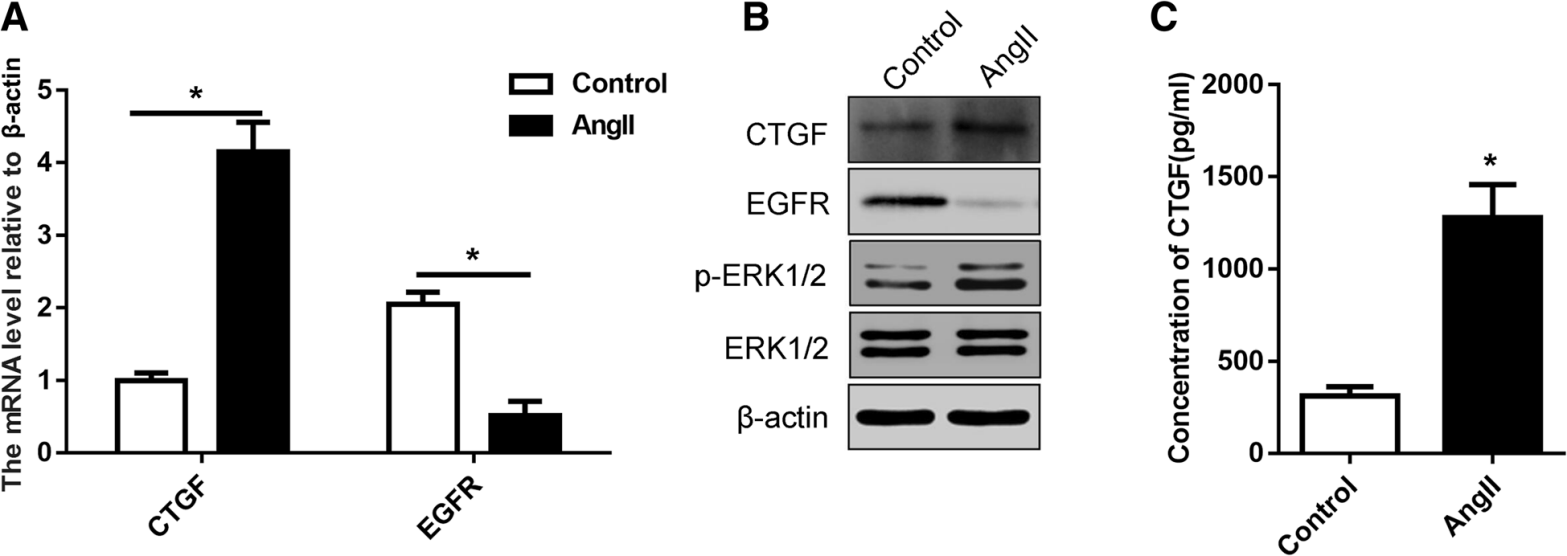
Ang-II stimulation promotes CTGF expression and reduces EGFR expression. **a** The mRNA expression of CTGF and EGFR in H9c2 cardiomyocytes with or without Ang-II treatment. **b** The expression of CTGF, EGFR, p-ERK1/2 and ERK1/2 were detected by western blot in H9c2 cardiomyocytes with or without Ang-II treatment. **c** The secretion of CTGF was detected by ELISA in medium of H9c2 cardiomyocytes with or without Ang-II treatment. All experiments were repeated at least three times. Data represent mean ± SD; * *P* < 0.05

**Fig. 3:**
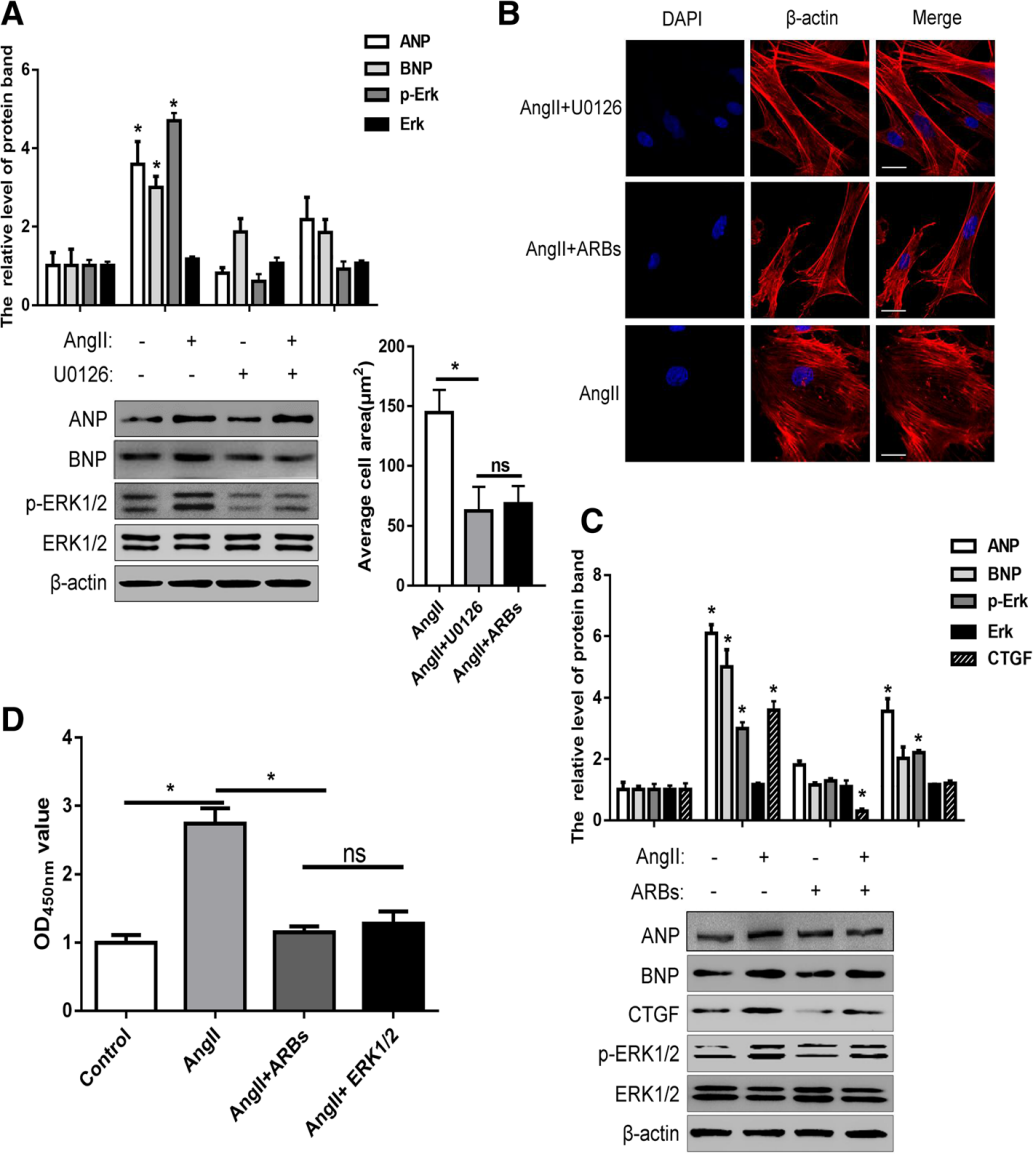
The expression of CTGF and EGFR under the stimulation of Ang-II in vivo. **a** The hematoxylin and eosin (HE)-staining (scale bar, 30 μm) of histological sections of the left ventricle in the indicated groups (*n* = 4 mice per experimental group). **b** The hematoxylin and eosin (HE)-staining (scale bar, 30 μm) of histological sections of the left ventricle in the indicated groups (*n* = 4 mice per experimental group). The immunohistochemistry assay shows expressions of CTGF and EGFR (brown) in the cytoplasm of cardiomyocytes (*n* = 4 mice per experimental group), scale bar, 30 μm. All experiments were repeated at least three times. Data represent mean ± SD; * *P* < 0.05

In a study by Andreas S. Barth et al. in 2006, a gene chip-based assay showed that high expression of CTGF was evident in 20 DCM cardiac tissues [14]. Also in a similar study in 2007, the results showed that EGFR showed low expression in DCM tissue [9]. Based on the above findings and our construction of the DCM cell model, we examined the expression of these protein molecules that may be involved in the regulation of DCM under the stimulation of Ang-II. Through qPCR and WB detection, both Ang-II-stimulated H9c2 cardiomyocytes exhibited increased CTGF and decreased EGFR at both mRNA and protein levels (Fig. 2a and b). Similarly, we also detected increased CTGF and decreased EGFR in the tissue of the rat cardiac hypertrophy model by immunohistochemistry (Fig. 3). Similarly, we detected the level of extracellular CTGF secreted by the ELISA assay, and the trend was consistent with the above results (Fig. 2c). In addition, considering the downstream signaling pathways regulated by EGFR, we also detected changes in the phosphorylation of ERK1/2, and as a result, the changes were in contrast to EGFR and consistent with CTGF. This also implies that there are other regulatory mechanisms between these signaling molecules and pathways.

### Ang-II stimulates activation of ERK1/2 signal and is mediated by GPCR

**Fig. 4:**
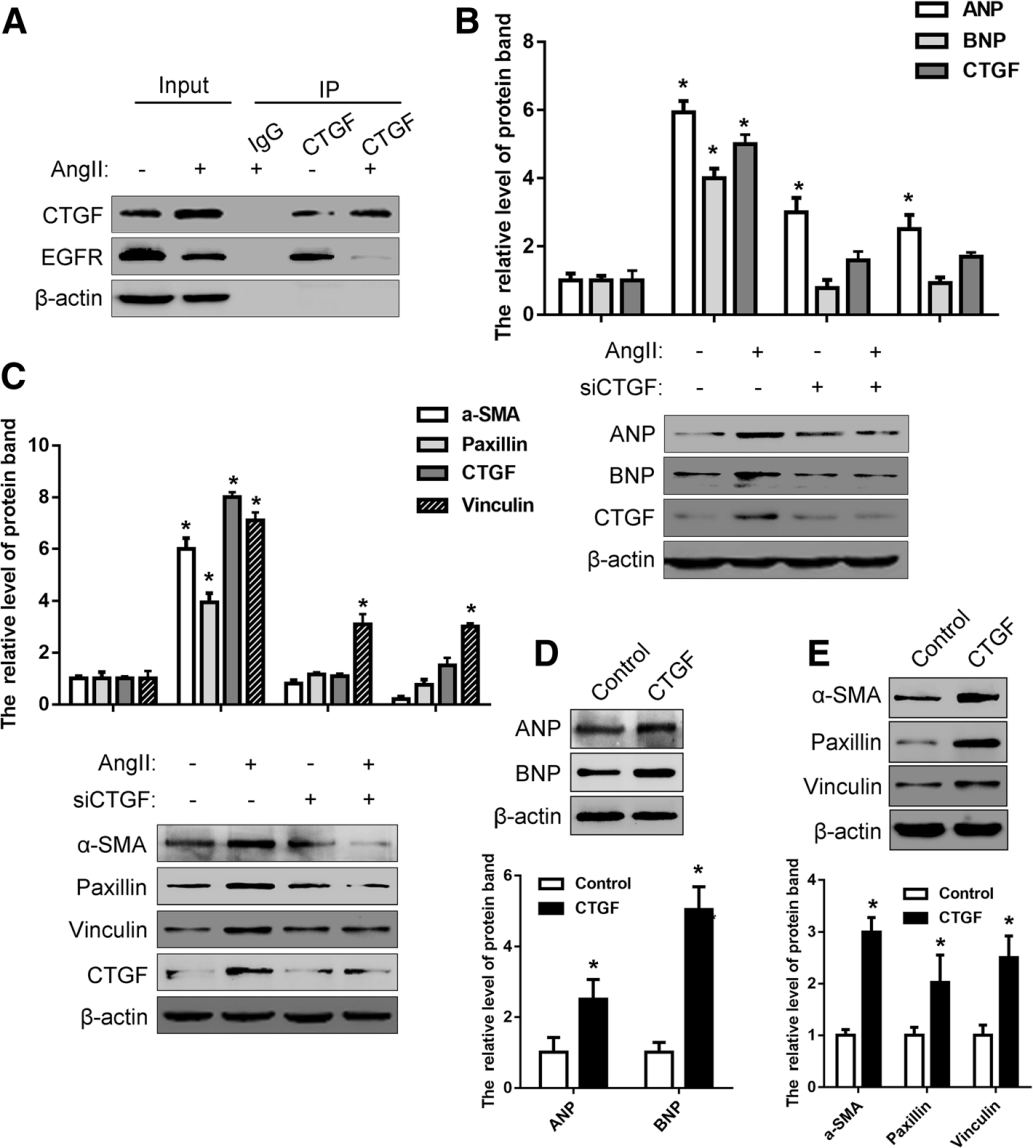
Ang-II stimulates activation of ERK1/2 signal and is mediated by GPCR. **a** The expression of ANP, BNP, p-ERK1/2 and ERK1/2 were detected by Western blot in H9c2 cardiomyocytes with or without Ang-II or U0126 treatment. **b** 24 h after seeding H9c2 cardiomyocytes in 10-cm culture plates, 10^− 8^ M AngII was added first with or without U0126 or ARBs treatment. And, the β-actin of H9c2 cardiomyocytes was immunofluorescent stain. **c** The expression of ANP, BNP, CTGF, p-ERK1/2 and ERK1/2 were detected by western blot in H9c2 cardiomyocytes with or without Ang-II or ARBs treatment. **d** The cell viability of H9c2 cardiomyocytes was detected using CCK-8 assay after treatment with Ang-II, ARBs or U0126. All experiments were repeated at least three times. Data represent mean ± SD; * *P* < 0.05

In view of the above results, we studied the ERK1/2 signaling activity triggered by Ang-II stimulation using U0126, an inhibitor of ERK1/2 signal. When U0126 was not added, Ang-II stimulation induced a significant increase in the amount of p-ERK1/2 protein, that is, the ERK1/2 signaling pathway was activated. And when U0126 was added, this phenomenon was eliminated (Fig. 4a). This further confirms that ERK1/2 appears to be active in the Ang-II-induced DCM cell model and also suggests that it plays an important role in the progression of DCM.

For further experiments, we used U0126 and Ang-II receptor inhibitors ARBs, respectively, to detect changes in cellular hypertrophy by actin staining. Under U0126 or ARBs treatment, Ang-II stimulation did not result in hypertrophy in H9c2 cardiomyocytes, which was significantly different from the Ang-II-stimulated hypertrophic model control group (Fig. 4b). Similarly, western blotting results also showed that Ang-II stimulation did not increase the expression of ANP and BNP when treated with U0126 or ARBs (Fig. 4a and c). At the same time, we also noted that Ang-II stimulation did not increase p-ERK1/2 in the presence of ARBs, and that the increased Ang-II-induced CTGF expression disappeared (Fig. 4c). These results indicate that increased expression of p-ERK1/2 and CTGF is regulated by Ang-II receptor, a GPCR. In addition, cell viability experiments showed that the downstream signal of p-ERK1/2 promotes cell activity, and high cell activity represents an increase in cell proliferation (Fig. 4d). The balance of proliferation and apoptosis of cardiomyocytes is also closely related to the occurrence and development of cardiac hypertrophy.

### Reduced interaction between CTGF and EGFR stimulated by Ang-II

**Fig. 5:**
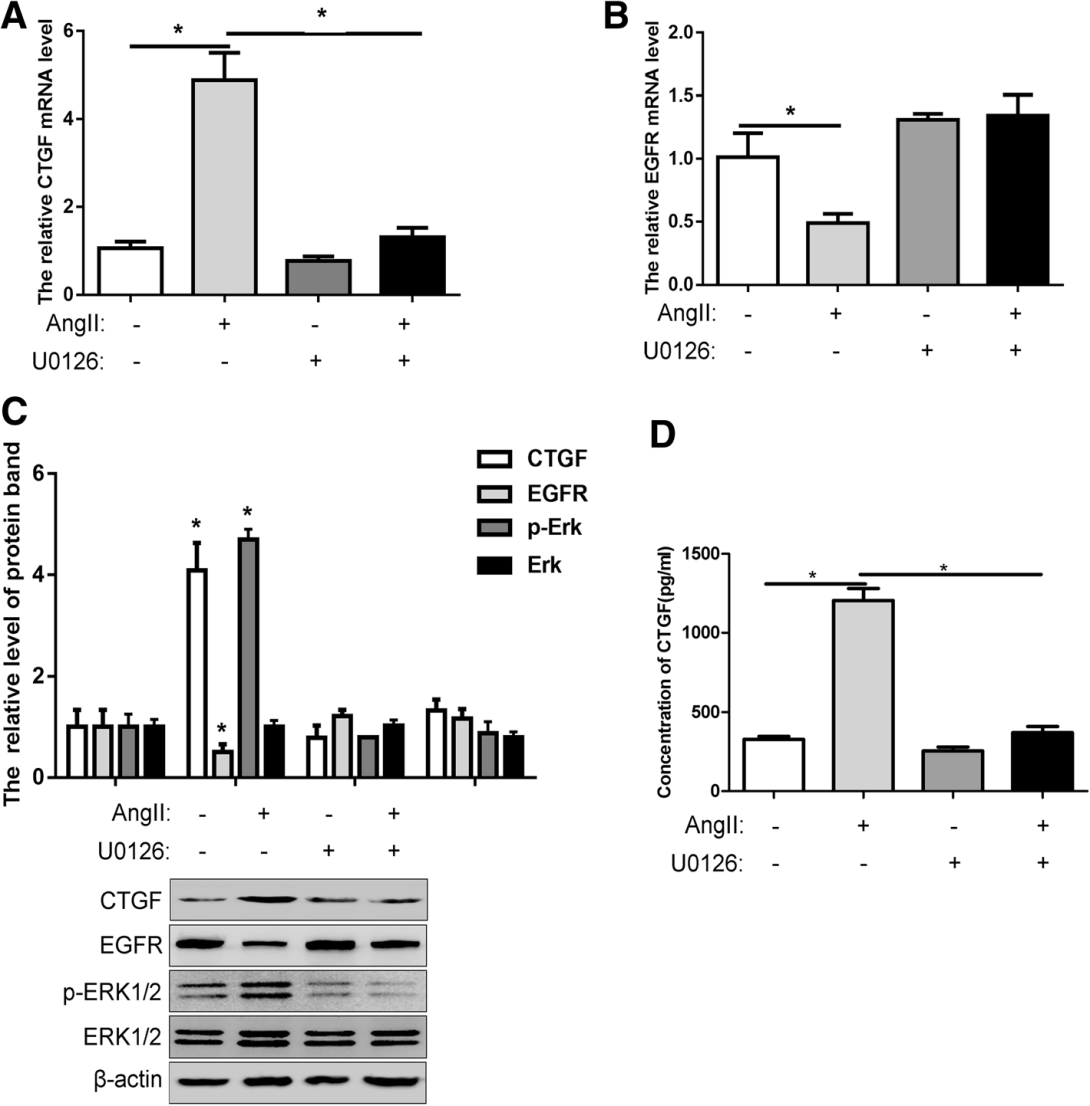
Reduced interaction between CTGF and EGFR stimulated by Ang-II. **a** The interaction of CTGF and EGFR was detected by co-IP in H9c2 cardiomyocytes with or without Ang-II treatment. **b** H9c2 cardiomyocytes were transfected with siCTGF for 24 h and then treated with or without Ang-II to detect the expression of ANP, BNP and CTGF by western blot. **c** H9c2 cardiomyocytes were transfected with siCTGF for 24 h and then treated with or without Ang-II to detect the expression of α-SMA, Paxillin and Vinculin by western blot. **d** H9c2 cardiomyocytes were treated with or without CTGF and then detected the expression of ANP and BNP by western blot. **e** H9c2 cardiomyocytes were treated with or without CTGF and then detected the expression of α-SMA, Paxillin and Vinculin by western blot. All experiments were repeated at least three times

As shown in Fig. 2, H9c2 cardiomyocytes stimulated by Ang-II exhibited significant high expression of CTGF and low expression of EGFR. We explored the relationship between them through further experiments. Co-immunoprecipitation experiments showed that there is an interaction between CTGF and EGFR, and this interaction is reduced with Ang-II stimulation (Fig. 5a). In addition, Ang-II stimulation did not increase the expression of ANP and BNP in the presence of siCTGF (Fig. 5b). Similarly, after the treatment with siCTGF, the increase of cell fibrosis induced by Ang-II stimulation disappeared, and the expression of fibrosis markers did not change significantly (Fig. 5c). In view of this, we also stimulated cells with recombinant synthetic CTGF, and the markers associated with cellular hypertrophy and fibrosis were significantly elevated (Fig. 5d and e). These results indicate that the interaction between CTGF and EGFR is reduced during the cardiac hypertrophy, while the former plays an important role.

### The expression of CTGF and EGFR under the stimulation of Ang-II is affected by the activity of ERK1/2

**Fig. 6:**
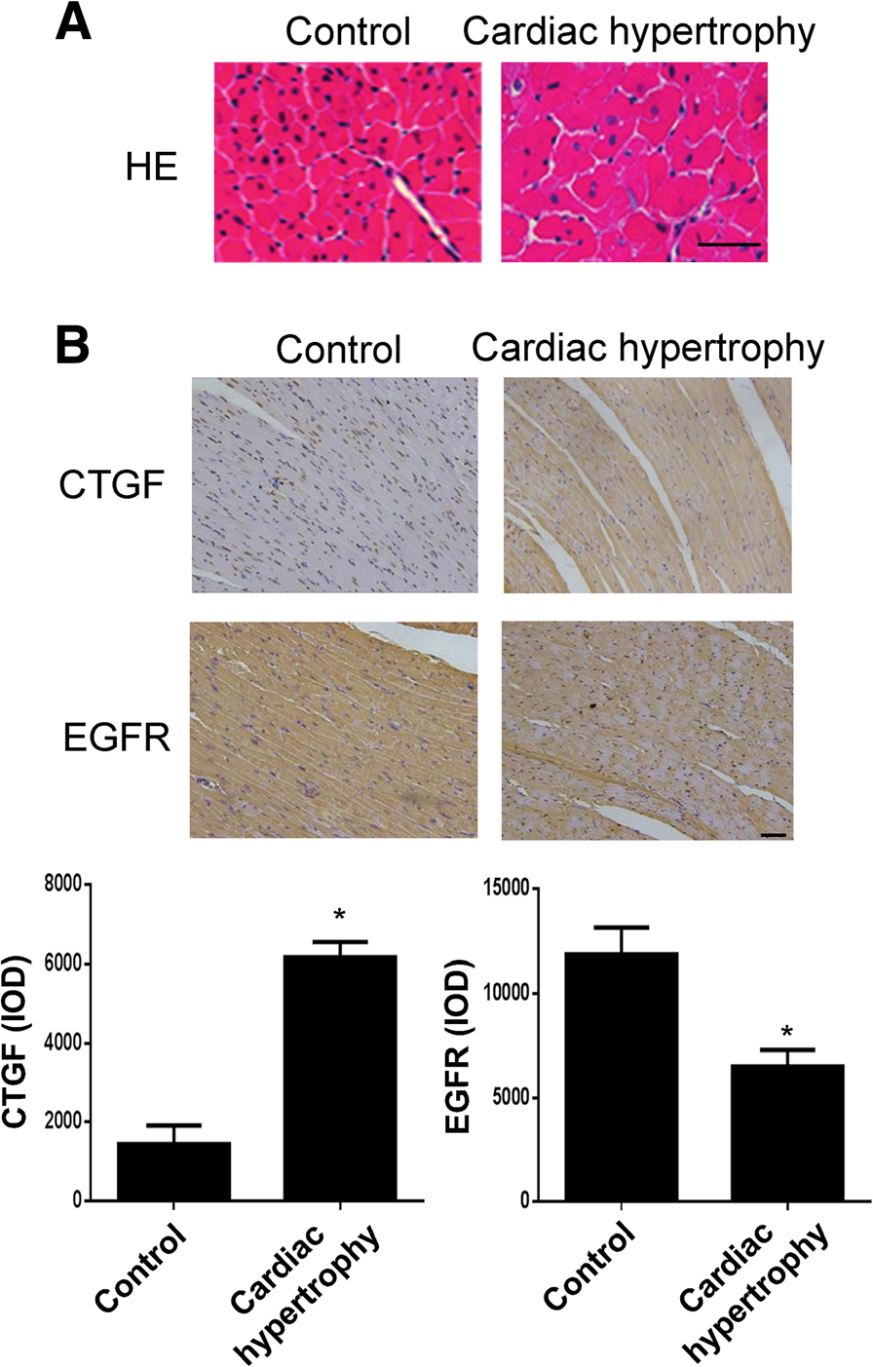
The expression of CTGF and EGFR under the stimulation of Ang-II is affected by the activity of ERK1/2. **a** The mRNA expression of CTGF or (**b**) EGFR were detected by RT-PCR in H9c2 cardiomyocytes with or without Ang-II or U0126 treatment. **c** The expression of CTGF, EGFR, p-ERK1/2 and ERK1/2 were detected by western blot in H9c2 cardiomyocytes with or without Ang-II or U0126 treatment. **d** The secretion of CTGF was detected by ELISA in medium of H9c2 cardiomyocytes with or without Ang-II or U0126 treatment. All experiments were repeated at least three times. Data represent mean ± SD; * *P* < 0.05

The changes in mRNA levels, protein levels, and extracellular secretion levels of CTGF under U0126 treatment were examined. The results showed that U0126 significantly inhibited the increase of CTGF induced by Ang-II stimulation (Fig. 6a and b). At the same time, the decrease in EGFR expression was also suppressed (Fig. 6c and d). These results indicate that ERK1/2 signaling regulates the expression of CTGF and EGFR, and that the regulation of CTGF expression will also affect the downstream cellular events that regulate it. Once again, it implied that the ERK1/2 signaling pathway may play an important role in the development of cardiac hypertrophy.

## Discussion

DCM is a kind of cardiomyopathy, and it occurs along with pathological phenomena such as cardiac hypertrophy, myocardial fibrosis and cardiomyocyte activity [1]. In response to this disease, a series of molecular mechanisms were studied based on the cell model of myocardial hypertrophy and fibrosis. The main results are as follows: 1) During the cardiac hypertrophy induced by Ang-II stimulation, CTGF was highly expressed and EGFR was significantly down-regulated; 2) Increased CTGF and decreased EGFR were regulated by ERK1/2 signaling pathway; 3) CTGF interacted with EGFR, and the interaction is reduced with the stimulation of Ang-II; 4) ERK1/2 serves as the center of signal regulation. On the one hand, the signal of AngII receptors is carried by ERK1/2, on the other hand, it regulates the expression of EGFR and plays a role in cooperative regulation.

The high expression of CTGF in DCM tissue has been reported in many previous studies [13, 14]. However, few of the specific molecular mechanisms underlying this pathological condition have been mentioned. Studies have shown that CTGF plays an important role in the process of ECM responses. Studies have also shown that CTGF significantly improved left ventricular (LV) systolic and diastolic function in PKCε mice, and slowed the progression of LV dilatation [3]. These studies have shown that CTGF plays an important role in the development of cardiac hypertrophy. It is in view of this that we have studied the upstream molecular signals that regulate CTGF overexpression. The experimental results demonstrate that CTGF is highly expressed in response to Ang-II stimulation, and that this change in expression is regulated by the ERK1/2 signaling pathway. This also indirectly shows that the ERK1/2 signaling pathway plays a key regulatory role in the development of cardiac hypertrophy.

As early as 1999, studies have found that CTGF can interact with TGFβ [15]. In the 2013 study, researchers demonstrated that CTGF can bind to EGFR and inhibit EGFR-mediated downstream phosphorylation events [16]. However, no studies have reported the changes and effects of their combination in the cardiomyopathy model. On the one hand, our study also demonstrated that CTGF and EGFR can interact with each other in a cardiomyocyte model. On the other hand, in view of the differential expression of CTGF and EGFR under Ang-II stimulation, we also demonstrated that this interaction is regulated by Ang-II. Ang-II stimulation promotes the expression of CTGF through the activation of ERK1/2 signaling pathway, while the expression of EGFR decreases. Increased expression of CTGF promotes the development of cellular hypertrophy, while a decrease in EGFR reduces the interaction with CTGF to release more CTGF to promote the development of cardiac hypertrophy.

ERK1/2 plays an important role in the occurrence and development of tumors as an important signaling pathway molecule and phospho-kinase in cells [17]. There are many researches in this area. In these studies, most of them are ERK1/2 that receives tyrosine kinase receptors signals, which in turn activate [18, 19]. Our study found that Ang-II stimulation can activate ERK1/2 signaling, which is a type of GPCR. At the same time, ERK1/2 feedback inhibited EGFR expression. This provides the possibility for ERK1/2 to coordinate GPCR and EGFR signaling during the cardiac hypertrophy. Our experimental results also confirmed this possibility, and this regulatory pattern directly regulates the expression and mode of action of downstream CTGF. This further expands the importance of ERK1/2 in the development of DCM disease.

## Conclusions

In here, we have demonstrated the ERK1/2 signaling pathway cooperates with GPCR and EGFR signaling, and promotes the occurrence and development of cardiac hypertrophy by regulating the expression and binding states of CTGF and EGFR in the cell model induced by Ang-II stimulation. In addition to confirming the role of CTGF during cardiac hypertrophy and possible therapeutic targets, this study also revealed a coordinated regulation model based on ERK1/2, suggesting that ERK1/2 signaling pathway may be an important control link for mitigation of cardiac hypertrophy treatment.

## Methods

### Cell line and cell culture

The H9c2 rat cardiomyocytes were purchased from the ATCC Biological Resources Center. After resuscitation cells were cultured in Dulbecco^,^ s modified Eagle^,^ s medium (DMEM) containing 10%FBS (Gibco, Life Technologies), 100 U/ml penicillin, and 100 mg/ml streptomycin. The cells were incubated in a humidified atmosphere of 95% air and 5% CO2 at 37 °C.

### Plasmids and siRNAs

Full-length EGFR was amplified from cDNA. The polymerase chain reaction (PCR) products were cloned into the pCMV-N-Flag (Beyotime, Nantong, China). The cells were seeded in 6-well plates, cultured to 80~90% confluence, and then transiently transfected with the plasmid by using Lipofectamine 3000 (Invitrogen) according to the reverse transfection method provided by the manufacturer.

Duplex oligonucleotides were chemically synthesized and purified by GenePharma (Shanghai, China). The small interfering RNA (siRNA) duplexes used were CTGF, #1, 5′- CACCGCAATACCTTCTGCAGGCTGGACAAGAGATCCAGCCTGCAGAAGGTATTGTTTTTTG-3′. Cells were transfected with siRNA duplexes using Lipofectamine 3000 (Invitrogen) according to the reverse transfection method provided by the manufacturer.

### Real time-qPCR

The collected cells were prepared for total RNA extraction using using Trizol reagent (Invitrogen, USA). The cDNAs were synthesized using a reverse transcription kit (Quant One Step RT-PCR kit, TIANGEN, China), and real-time PCR was performed using SYBR green master mix (Quant one step qRT-PCR Kit, TIANGEN, China) on a MyiQTM2 (BIORAD, USA). β-actin was used to normalize the real-time PCR data. The following primer sequences were used: CTGF, 5′- GGCCTCTTCTGCGATTTCG-3'and 5′-GGCCTCTTCTGCGATTTCG-3′; EGFR, 5′-GGCCTCTTCTGCGATTTCG-3′ and 5′-GCAGCTTGACCCTTCTCGG-3′;β-actin, 5′-ATGGAGGGGAATACAGCCC-3′ and 5′-TTCTTTGCAGCTCCTTCGTT-3′.

### Western blotting

The cells were lysed in radioimmune precipitation assay (RIPA) buffer supplemented with protease inhibitor cocktail (Roche, Shanghai, China) to prepare the protein sample. Protein concentration was measured using Enhanced BCA Protein Assay Kit (Beyotime Biotechnology, China) and 40 μg of protein were loaded and separated by 10% sodium dodecyl sulfate polyacrylamide gel electrophoresis. Proteins were transferred to PVDF membranes and then blocked with 5% BSA in Tris-buffered saline with Tween-20 (TBST) before immunodetection with the following antibodies: BNP (1:1000, CST, USA), ANP (1:1000, CST, USA), Paxillin (1:1000, CST, USA), Vinculin (1:1000, CST, USA), CTGF (1:1000, CST, USA), ERK (1:1000, CST, USA), p-ERK (1:1000, CST, USA), β-actin (1:1000, CST, USA). After Primary antibody incubation for 16 h, the PVDF membranes were washed by TBST buffer before incubated with secondary antibody for 1.5 h. Specific binding was visualized by ECL reaction. The western blot bands were quantified using Image J Software (version 1.41).

### Immunofluorescent staining

Cells were fixed with 4% paraformaldehyde (PFA). Then, the cells were incubated with primary antibody against β-actin, followed by washing and incubation with AF594-conjugated goat anti-rabbit secondary antibodies (1:250; Earthox, USA) for 2 h at 37 °C. The nuclei were stained with 4′, 6-diamidino-2-phenylindole (DAPI). Fluorescent images were visualized and captured using an inverted fluorescence microscope (Olympus, Tokyo, Japan). Images were visualized and captured using a phase contrast microscope (Olympus, Tokyo, Japan).

### Cell viability assay

Cells were seeded in a 96-well plate as a density of 5000 cells/well for 24 h. Cell viability was measured using the Cell Counting Kit-8 (Dojindo Molecular Technologies, Japan), according to the manufacturer’s instructions. Absorbance was determined using the Multi-Mode Microplate Reader (PerkinElmer, Finland).

### ELISA assay

Cells culture supernatant was prepared for CTGF (Mouse CTGF ELISA Kit, Abcam, Cambridge, UK) detection according to the manufacturers’ protocol.

### Statistical analysis

Data are presented as means ± SD. Statistical comparisons were performed by either Student’s 2-tailed t test or ANOVA with Tukey multiple comparison post-test, as appropriate. A *P* value of less than 0.05 was considered significant.

## Data Availability

The data that support the findings of this study are available from the corresponding author upon reasonable request.

## Abbreviations

Ang-II: Angiotensin II
ANP: Atrial natriuretic peptide
ARBs: Angiotensin II receptor blockers
BNP: B-type natriuretic peptide
CTGF: Connective tissue growth factor
DAPI: 4′, 6-diamidino-2-phenylindole
DCM: Dilated cardiomyopathy
EGFR: Epidermal growth factor receptor
ELISA: Enzyme linked immunosorbent assay
EMT: Epithelial-mesenchymal transitions
ERK1/2: Extracellular regulated protein kinase 1/2
GPCR: G protein coupled receptor
PCR: Polymerase chain reaction
RTK: Receptor tyrosine kinase
siRNA: small interfering RNA
TBST: Tris-buffered saline with Tween-20
